# Supplementary material for: Association between oral microbiome and seven types of cancers in East Asian population: a two-sample Mendelian randomization analysis
Source: Front Mol Biosci. 2023 Nov 21;10:1327893. doi: 10.3389/fmolb.2023.1327893 (PMC10702768; doi:10.3389/fmolb.2023.1327893)
Supplement: Supplementary file 1 [file DataSheet1.ZIP › Supplementary Materials/MR plots for tongue/tongue═╝/Lung cancer/pheno.3150_to_lung cancer_forest.pdf]

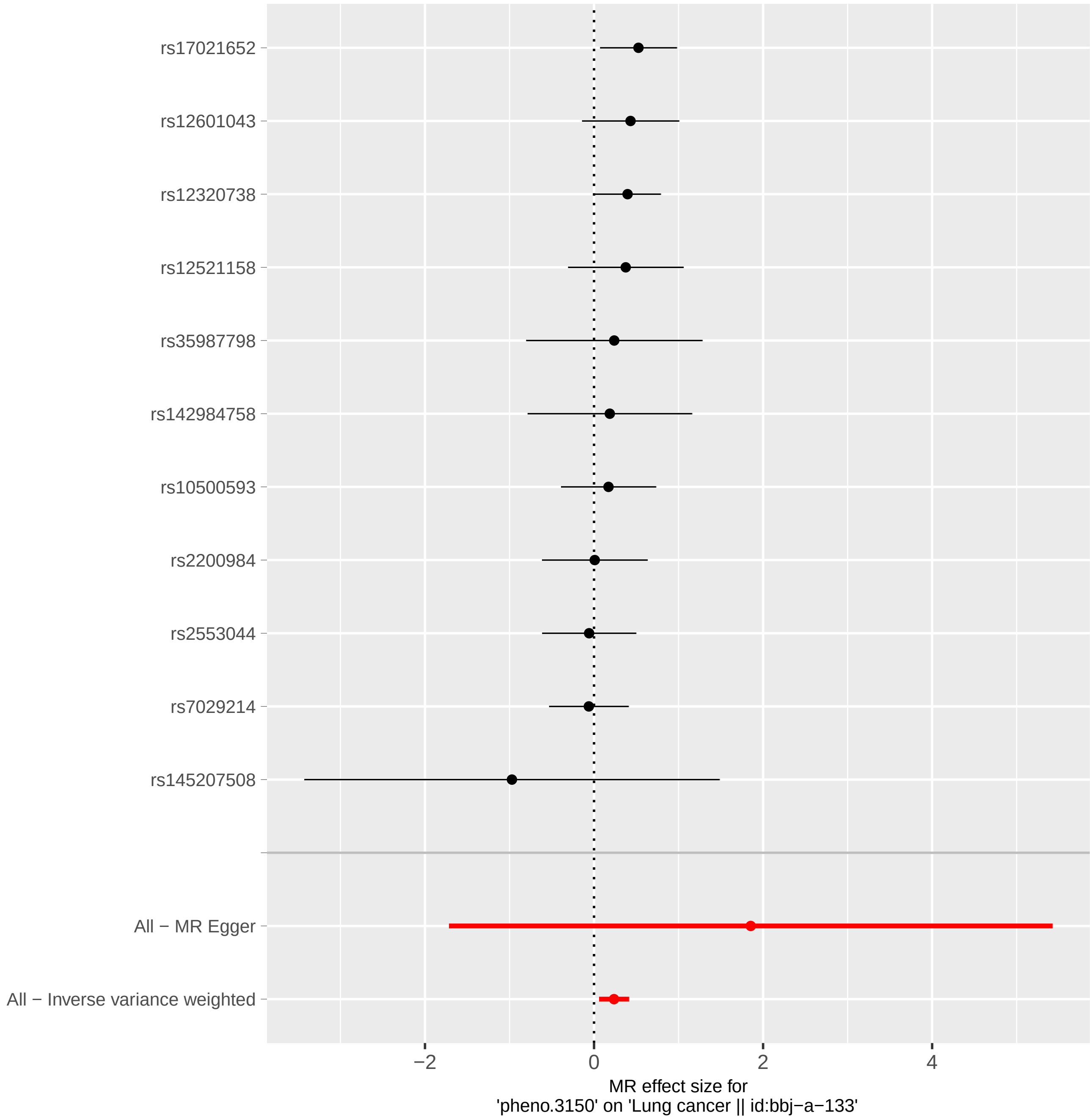
rs17021652
rs12601043
rs12320738
rs12521158
rs35987798
rs142984758
rs10500593
rs2200984
rs2553044
rs7029214
rs145207508
All − MR Egger
All − Inverse variance weighted
−2
0
2
4
MR effect size for
'pheno.3150' on 'Lung cancer || id:bbj−a−133'
